# Risk Assessment of Hydrogen-Powered Aircraft: An Integrated HAZOP and Fuzzy Dynamic Bayesian Network Framework

**DOI:** 10.3390/s25103075

**Published:** 2025-05-13

**Authors:** Xiangjun Dang, Yongxuan Shao, Haoming Liu, Zhe Yang, Mingwen Zhong, Huimin Zhao, Wu Deng

**Affiliations:** 1 School of Safety Science and Engineering, Civil Aviation University of China, Tianjin 300300, China; 2 Tianjin Aviation Equipment Safety and Airworthiness Technology Innovation Center, Tianjin 300300, China; 3 School of Electronic Information and Automation, Civil Aviation University of China, Tianjin 300300, China; hmzhao@cauc.edu.cn

**Keywords:** hydrogen-powered aircraft, risk assessment, hazard and operability analysis, dynamic Bayesian network, fuzzy theory

## Abstract

To advance the hydrogen energy-driven low-altitude aviation sector, it is imperative to establish sophisticated risk assessment frameworks tailored for hydrogen-powered aircraft. Such methodologies will deliver fundamental guidelines for the preliminary design phase of onboard hydrogen systems by leveraging rigorous risk quantification and scenario-based analytical models to ensure operational safety and regulatory compliance. In this context, this study proposes a comprehensive hazard and operability analysis-fuzzy dynamic Bayesian network (HAZOP-FDBN) framework, which quantifies risk without relying on historical data. This framework systematically maps the risk factor relationships identified in HAZOP results into a dynamic Bayesian network (DBN) graphical structure, showcasing the risk propagation paths between subsystems. Expert knowledge is processed using a similarity aggregation method to generate fuzzy probabilities, which are then integrated into the FDBN model to construct a risk factor relationship network. A case study on low-altitude aircraft hydrogen storage systems demonstrates the framework’s ability to (1) visualize time-dependent failure propagation mechanisms through bidirectional probabilistic reasoning, and (2) quantify likelihood distributions of system-level risks triggered by component failures. Results validate the predictive capability of the model in capturing emergent risk patterns arising from subsystem interactions under low-altitude operational constraints, thereby providing critical support for safety design optimization in the absence of historical failure data.

## 1. Introduction

The continuous emission of greenhouse gases may lead to global climate disasters, and the development of green aviation is of great significance in stimulating the development of the green economy and alleviating carbon emission pressures [1,2]. Hydrogen-powered low-altitude aircraft, which do not emit carbon dioxide during operation, only necessitate consideration of the impacts of NOx and water vapor on the atmosphere [3]. In addition, the development of low-altitude aircraft is expected to alleviate the immense pressure on current ground transportation [4,5,6,7,8]. Consequently, hydrogen-powered low-altitude aircraft have emerged as an important research direction in the development of green aviation.

In 2020, NASA conducted a survey to assess public acceptance of electric vertical takeoff and landing (eVTOL) aircraft, revealing that individuals would be willing to embrace this new mode of transportation provided it meets sufficient safety satisfaction [9]. However, hydrogen poses inherent safety risks due to its wide flammability range (4–75%) and extremely low minimum ignition energy (the minimum ignition energy of hydrogen in the air is 0.017 mJ), making it prone to fire or explosion upon contact with sparks in case of leakage. Most existing research focuses on the operational safety of air traffic [10,11,12,13,14] and the design methodologies of aircraft [15,16,17,18,19,20], with limited attention given to the safety of the aircraft itself under operational modes. Therefore, accurately identifying and precisely assessing the risks associated with onboard hydrogen systems is a critical prerequisite for overcoming the developmental constraints. The onboard environment is complex and dynamic, with the onboard hydrogen-powered system integrating hydrogen-powered units and battery units capable of signal interaction. During the design process, reliable risk assessment can provide essential references for system optimization and iteration, thereby enhancing system safety. The onboard hydrogen systems of hydrogen-powered low-altitude aircrafts present numerous potential safety hazards [21], necessitating efficient and accurate risk assessments to provide safety assurance limits for its design, production, operation, and maintenance phases. However, onboard hydrogen systems possess complex and dynamic characteristics, where failures in one component can affect other components in complex ways. The interactions among components, as well as the interactions between the system and its environment, can lead to the propagation and evolution of these failures over time [22,23,24]. Furthermore, current research on hydrogen-powered low-altitude aircraft remains theoretical and lacks engineering experience data to support quantitative risk assessments. Therefore, risk assessment methods for onboard hydrogen systems must be capable of capturing all risk diffusion pathways and accurately quantifying the dynamic process of risk evolution in situations of insufficient data. In summary, risk assessment methods for onboard hydrogen systems must fulfill the following capabilities [25,26,27,28].

- Identify risk factors;
- Complete insufficient data or replace uncertain data;
- Reflect the evolution of uncertainties of risk factors over time;
- Quantify the relationships of uncertainty among risk factors.

Hazard and operability analysis (HAZOP) and Bayesian network (BN) are two knowledge driven methodologies frequently employed in risk assessment [29,30,31,32,33,34].

Compared with other risk assessment methods, HAZOP is adept at accurately identifying the causes of dynamic risk changes [35], it performs risk analysis by recognizing process deviations and capturing the causal events associated with these deviations. Therefore, HAZOP is widely applied in the assessment of system risks. Giardina et al. [36] developed the FHIA method, which combines FMECA and HAZOP. Their study applied the FHIA method to analyze risks in LNG storage systems. The results demonstrated that this approach effectively identifies potential human error factors, causal factors in faults, multiple or common cause failures, as well as the cause–consequence correlations of hazards at different stages of the process. Oh et al. [37] utilized HAZOP to analyze the risk situation with different installation positions of the pressure safety valve in the supply tank, concluding that the pressure safety valve does not need to be installed at the top of the supply tank. Shen et al. [30] employed HAZOP and FMEA methods to identify potential accident scenarios related to the onboard hydrogen storage and supply system. Furthermore, by comparing the risk matrix with and without the implementation of safety measures, they concluded that implementing safety measures could reduce the risks associated with onboard hydrogen storage and supply systems to an acceptable range. Jouber et al. [31] designed a safety assessment method for a large bulk material dismantling system based on HAZOP, using guide words specifically tailored for this system and incorporating lessons learned into the analysis process, they verified that the results of this method are more accurate and reliable.

Bayesian network (BN) is a method that combines graphical representations with probability theory to represent probabilistic relationships between variables and to address uncertainty relationships [32,38,39]. The network graphic structure consists of nodes and directed edges, where the nodes represent risk factors, and the directed edges represent the influence relationships between them. Probability theory is based on the Bayesian principle, which gives BNs the ability to perform bidirectional reasoning.

BNs can construct complex networks of influence relationships among risk factors and quantify the uncertainty relationship of risk factors, making them widely applicable in the field of risk analysis [40]. Jafari et al. [33] constructed a theoretical model for evaluating the risk of explosions in chemical plants based on BNs and provided an overall evaluation of the risk of explosions under different scenarios. Rathnayaka et al. [34] used the event tree method for qualitative analysis of accident scenarios and mapped the results to BNs model to obtain risk assessment results for various accident scenarios. Liu et al. [41] conducted fault diagnosis for a solar assisted heat pump based on BN. They determined the parameters of the BN from incomplete data using the BP neural network and maximum likelihood estimation, and they estimated the parameters from incomplete expert knowledge using the BP neural network and fuzzy set theory. Their study analyzed multiple cases, and the results demonstrated that the BN can perform fault diagnosis with both complete and incomplete symptom data. Ahmadisourenabadi et al. [42] proposed a BN-based risk assessment method, employing an improved lexicographic augmented-constraint method to optimize three objective functions: cost, pollution, and resilience. This model can be effectively used to quantify and enable the resiliency of a microgrid.

However, BN have the following limitations [43,44]:

Limitation 1: When data are insufficient or uncertain, the construction of BNs may be incomplete. Therefore, the use of BNs for risk assessment requires relying on complete engineering experience data.

Limitation 2: The uncertain relationships between nodes do not change over time, thus failing to capture the dynamic characteristics of risk variations.

Traditional BNs can be extended into dynamic Bayesian networks (DBN) and fuzzy Bayesian networks (FBN). DBN is a temporal extension of traditional BN, capable of revealing the evolution of uncertainty relationships of risk factors over time [45], thus addressing limitation 2 of BN. FBN is a combination of traditional BN and fuzzy set theory [46], enabling the resolution of issues related to incomplete model construction when accurate data sources are lacking, thereby addressing limitation 1 of BN.

Fuzzy set theory identifies the membership set of uncertain factors through their values of membership functions, and it is commonly used to deal with uncertainty issues [47]. Similarity aggregation method (SAM) is an expert evaluation integration method based on fuzzy set theory, capable of resolving the issue of converting expert opinions when there are significant differences among them [48], and it is widely applied in FBN.

Fuzzy dynamic Bayesian network (FDBN) effectively integrates DBN with FBN [49], incorporating fuzzy set theory to address missing and anomalous data within the DBN framework. It combines the advantages of DBN and FBN, enabling it to achieve data source completeness through fuzzy set theory and to quantitatively demonstrate the dynamic variation patterns of uncertain relationships among risk factors under the complex interactions of system components. Therefore, FDBN can overcome the limitations 1 and 2 of BN. In addition, the structure and parameters of FDBN play a role for performance. Many authors have provided some different optimization algorithms [50,51,52,53,54,55,56,57], which can be used to optimize the network structure and parameters.

Using the risk assessment capability requirements of onboard hydrogen systems as the criteria for evaluation, HAZOP can adeptly “identify risk factors”, while the FDBN method excels with “complete insufficient data or replace uncertain data”, “reflect the evolution of risk factors and their uncertainties over time”, and “quantify the relationships of uncertainty among risk factors”. Therefore, the combination of HAZOP and FDBN can fulfill all requirements for quantitative risk analysis of onboard hydrogen systems.

Based on the above discussion, risk assessment is a critical component in the development of onboard hydrogen systems for hydrogen-powered aircraft. Existing research methods often fail to fully address the need for comprehensive risk assessment of complex, dynamic onboard hydrogen systems, especially when data are insufficient or uncertain. To address this challenge, this paper proposes a new methodology combining HAZOP with FDBN, referred to as HAZOP-FDBN quantitative risk assessment method.

The method is based on HAZOP results, taking safety-affecting process deviations as the starting point to qualitatively capture system risk factors. Using these results as inputs, it reorganizes them based on the paths of risk diffusion to obtain a topological network structure of risk diffusion. Through fuzzy processing based on expert experience, it acquires the probabilistic foundation for dynamic risk behaviors. By matching the topological network structure with the probabilistic foundation using FDBN, it constructs a dynamic risk evolution assessment model. Based on the model’s computational results, the method completes the risk assessment quantitatively and formulates corresponding safety constraints.

The results based on HAZOP-FDBN contribute to a comprehensive risk assessment of the onboard hydrogen system. The methodology addresses the limitations of traditional risk assessment methods that are unable to identify risk diffusion pathways and quantitative risk assessment that is highly dependent on the completeness and accuracy of data sources. The risk assessment results derived from this methodology provide scientifically based and feasible recommendations for the design, production, utilization, and maintenance of hydrogen-powered aircraft.

The main contributions of this study are listed as follows:

- The proposed method achieves the transition between qualitative and quantitative analyses while maintaining risk assessment boundaries by leveraging the characteristics of HAZOP and FDBN methodologies. It deconstructs HAZOP results and maps them into risk propagation paths based on causal relationships. By establishing conversion rules between risk propagation paths and risk diffusion networks, it realizes the network-based representation of risk dynamic evolution process.
- A method is introduced to convert expert evaluations into input probabilities for FDBN-based risk diffusion networks using fuzzy set theory. Dynamic risk expert evaluation rules are established, clarifying the specific approaches for obtaining expert assessments and probabilities at different nodes.
- Taking the onboard hydrogen system of low-altitude aircraft as a case study, the HAZOP-FDBN risk assessment framework is applied to complete risk evaluation. Examples of safety constraint derivation are demonstrated based on partial assessment results.

## 2. Methodology

In the HAZOP-FDBN-based risk assessment framework, the primary methodologies involved include HAZOP, DBN, and the similarity aggregation method (SAM) for processing expert opinions based on fuzzy theory. This section provides an introduction to the fundamental concepts related to these methods.

### 2.1. HAZOP

HAZOP decomposes a system into subsystems with distinct functions, which serve as analysis nodes. These nodes are used to identify process deviations at each node that may cause unacceptable consequences and analyze their causes and consequences. The results of HAZOP are presented in a causal chain structure, encompassing all risk pathways associated with potential system functional abnormalities triggered by component failures. HAZOP is often utilized in risk assessment to determine the paths of risk propagation based on process deviations, the causes of these deviations, and their consequences. Process deviations refer to the extent to which a system’s state parameters deviate from the set values [58], causes of the deviations refer to the equipment or component failure event that leads to the occurrence of the deviation; consequences of the deviations are the direct outcome resulting from the propagation of the deviation.

### 2.2. DBN

BN [59] is a method based on graph theory and probability theory. The network structure is a directed acyclic graph composed of nodes and directed edges [60]. Nodes represent random variables, and directed edges indicate the influence relationships between variables, pointing from parent nodes to child nodes. Probabilistic networks are based on Bayesian principle as shown in Equation (1), serving as the theoretical foundation for describing the dependency relationships between nodes.

(1) $$P(A\left|B\right.)=\frac{P(A)P(B\left|A\right.)}{P(B)}$$

where $P(A\left|B\right.)$ is the posterior probability of *A*, P(A) is the prior probability of *A*, $P(B\left|A\right.)$ is the probability of likelihood, P(B) is the probability of failure for B.

**Figure 1:**
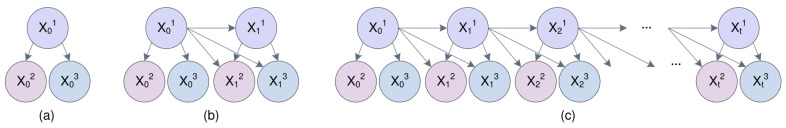
(**a**) DBN initial network (**b**) DBN transfer network (**c**) DBN representation diagram.

The DBN is an extension of the BN in the time dimension [61] and consists of an initial network and a transfer network, denoted by the symbol $\left(B_{0},B_{\to }\right)$. Here, $B_{0}$ denotes the initial network, which represents the BN model at the initial moment, as shown in Figure 1a. $B_{\to }$ denotes the transfer network, as shown in Figure 1b. The window of the DBN is divided into time slices (t=0,1,⋯,T), nodes on different time slices are connected using the transfer network, extending the BN model along the time axis, as shown in Figure 1c.

To construct the DBN model, the following two principles need to be followed:

- The states of each node at a given time slice are influenced only by the states of the node in the previous time slice.
- The conditional probability remains stable across all time slices.

The conditional probability distribution for neighboring time slices in the DBN model is described as follows:

(2) $$P(X_{t}\left|X_{t-1}\right.)=\prod_{i-1}^{n}P(X_{t}^{i}\left|Pa(X_{t}^{i})\right.)$$

where $X_{t}^{i}$ denotes the *i*th node on time slice *t*, $Pa(X_{t}^{i})$ denotes the parent node of node $X_{t}^{i}$, n is the number of nodes in the network.

The joint probability density function of DBN from time slice *t* = 1 to *T* is shown in Equation (3).

(3) $$P(X_{1:T})=\prod_{t=1}^{T}\prod_{i=1}^{n}P(X_{t}^{i}\left|Pa(Z_{t}^{i})\right.)$$

### 2.3. SAM

SAM is a fuzzy theory-based approach that transforms expert evaluations into fuzzy data and is commonly used for constructing quantitative risk assessment data sources [62,63,64]. This method employs linguistic terms to define the language of expert evaluations and establishes expert evaluation criteria. Experts draw on their experience to assess the likelihood of an event occurring, integrating the evaluation results from various experts regarding the same event into fuzzy results. The SAM takes into account both the degree of variance in expert evaluations and the credibility of expert opinions, calculating overall fuzzy numbers to represent the aggregated fuzzy results. Overall fuzzy numbers require defuzzification to obtain risk probability values, which are utilized in the construction of data sources for quantitative risk assessment. The selection of the similarity aggregation method in this study primarily references the works cited as [65,66]. The following steps present a detailed introduction to the specific implementation steps.

Step 1: Expert evaluation converted into fuzzy set.

**Figure 2:**
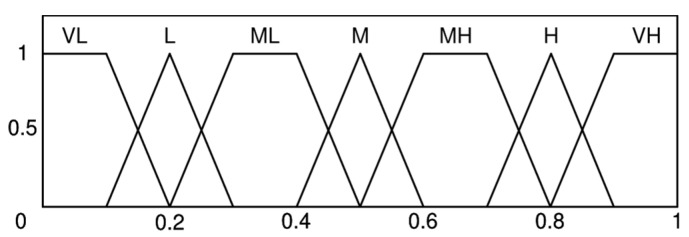
Membership functions.

Seven levels of linguistic terms are set for expert evaluation: very low (VL); low (L); mildly low (ML); medium (M); mildly high (MH); high (H); very high (VH). The membership functions required for this study are constructed by combining triangular membership functions and trapezoidal membership functions [51], as illustrated in Figure 2. Each linguistic value corresponds to a fuzzy set, which comprises four numerical values, as shown in Table 1.

Step 2: Calculate the agreement degree $S({\tilde{R}}_{u},{\tilde{R}}_{v})$ of the opinions between expert $E_{u}$ and expert $E_{v}$.

$S({\tilde{R}}_{u},{\tilde{R}}_{v})\in [0,1]$, the magnitude of $S({\tilde{R}}_{u},{\tilde{R}}_{v})$ reflects the degree of divergence between the opinions of the two experts, the closer it is to 1, the more aligned the experts’ opinions are. The calculation method of $S({\tilde{R}}_{u},{\tilde{R}}_{v})$ is shown in Equation (4).

(4) $$S({\tilde{R}}_{u},{\tilde{R}}_{v})=1-\frac{\sum_{i=1}^{4}\left|a_{i}-b_{i}\right|}{4},\;(v\neq u,u,v=1,2,\cdots ,n)$$

where ${\tilde{R}}_{u}=(a_{1},a_{2},a_{3},a_{4})$ and ${\tilde{R}}_{v}=(b_{1},b_{2},b_{3},b_{4})$ represent the fuzzy sets transformed according to the values evaluated by the two experts, respectively.

Step 3: Calculate the weighted agreement degree of the experts, denoted as $WA(E_{u})$.

The assignment of professional title, research duration in the relevant field, and age as criteria for assessing the credibility of expert evaluation results.

Experts with varying levels of experience may have different judgments regarding the same event. Therefore, assigning weights to experts is commonly adopted to reflect the differing reference values of their opinions. In this study, professional title, research duration in the relevant field, and age are used as criteria for assessing the credibility of expert evaluation results.

Professional title represents official recognition of experts’ capabilities and achievements. A senior professional title typically indicates that the expert possesses a systematic knowledge base, extensive practical experience, and high academic standing in their field.

Research duration in the relevant field directly reflects the depth of an expert’s experience. Those with long-term dedication to a particular domain are more likely to possess thorough understanding of its core issues, cutting-edge developments, and potential risks.

Age is often associated with professional career stages. Senior experts may have encountered a greater number of practical cases and tend to analyze complex issues more comprehensively.

Table 2 presents the weighting criteria and weight score of experts. Using the sum of weight scores to represent the authority of expert evaluation results.

Based on the information in Table 2, obtain the weight scores for each expert and calculate the weight value $W(E_{u})$ for expert $E_{u}$.

(5) $$W(E_{u})=\frac{S_{u}}{\sum_{u=1}^{n}S_{u}},\;(u=1,2,\cdots ,n)$$

where $S_{u}$ is the sum of weight score of expert $E_{u}$.

Substitute the expert weight values into the following formula to calculate the weighted agreement of the expert opinions. This step aims to correct the bias of expert opinions through their weight values, to reduce the evaluation result bias caused by expert expertise.

(6) $$WA(E_{u})=\frac{\sum_{v=1}^{n}W(E_{v})S({\tilde{R}}_{u},{\tilde{R}}_{v})}{\sum_{v=1}^{n}W(E_{v})},\;(v\neq u,u,v=1,2,\cdots ,n)$$

where $W(E_{u})$ and $W(E_{v})$ denote the weight values of experts $E_{u}$ and $E_{v}$, respectively.

Step 4: Calculate the relative agreement degree of the expert $E_{u}$, denoted as $RAD(E_{u})$.

(7) $$RAD(E_{u})=\frac{WA(E_{u})}{\sum_{u=1}^{n}WA(E_{u})},\;(u=1,2,\cdots ,n)$$

Step 5: Calculate the consensus degree coefficient of expert, denoted as $CDC(E_{u})$.

(8) $$CDC(E_{u})=\beta W(E_{u})+(1-\beta )RAD(E_{u})$$

where β is the relaxation factor, which β=0.5 is chosen in this study.

Step 6: Calculate the overall fuzzy number, denoted as $\tilde{R}$.

(9) $$\tilde{R}=\sum_{u=1}^{n}CDC(E_{u}){\tilde{R}}_{u}$$

Step 7: Calculate the fuzzy possibility score (FPS).

Using CoA defuzzification technique [67,68] to convert $\tilde{R}$ to FPS. Using FPS to represent the likelihood of a certain element’s failure.

(10) $$FPS=\frac{\int_{a}^{b}\frac{bx-a}{b-a}xdx+\int_{b}^{c}xdx+\int_{c}^{d}\frac{d-x}{d-c}xdx}{\int_{a}^{b}\frac{bx-a}{b-a}dx+\int_{b}^{c}dx+\int_{c}^{d}\frac{d-x}{d-c}dx}=\frac{1}{3}\frac{{\left(c+d\right)}^{2}-cd-{\left(a+b\right)}^{2}+ab}{c+d-a-b}$$

Step 8: Calculate the fuzzy failure probability (FFP).

Use the Onisawa function introduced in reference [69] to calculate FFP.

(11) $$FFP=\left\{\begin{array}{cc} \frac{1}{10^{2.301\times \left(\frac{1-FPS}{FPS}\right)}} & FPS\neq 0\\ 0 & FPS=0 \end{array}\right.$$

In the above steps, steps 1–6 outline the process of fuzzifying the expert evaluation results to obtain the aggregated outcome $\tilde{R}$. Step 7 and step 8 are the defuzzification process, which transform the expert evaluation fuzzy sets into FFP. FFP will be used as probability information to construct the DBN model.

## 3. Methodological Framework

**Figure 3:**
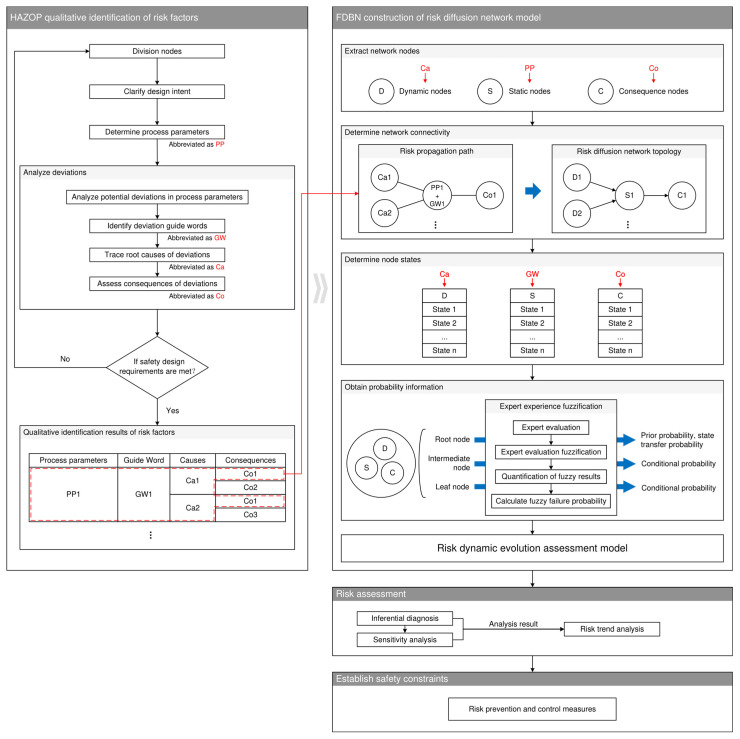
Risk assessment framework based on HAZOP-FDBN (Symbols in this figure are specific to this illustration).

The capture of risk factors and the identification and quantification of the dynamic process of risk propagation constitute fundamental requirements for risk assessment of onboard hydrogen systems. To fulfill these requirements, this section introduces a dynamic risk assessment framework integrating HAZOP and FDBN methodologies. The proposed framework comprises four key components: HAZOP-based qualitative identification of risk factors, FDBN-based construction of a risk diffusion network model, risk assessment and establish safety constraints as illustrated in Figure 3.

### 3.1. HAZOP Qualitative Identification of Risk Factors

The HAZOP method enables qualitative analysis to identify systemic risk factors. HAZOP results consist of process deviations, guide words, causes of deviations, and accident consequences. The acquisition methods are outlined in Table 3.

### 3.2. FDBN Construction of Risk Diffusion Network Model

FDBN modeling includes two main steps: (a) constructing the DBN graphical structure based on the HAZOP results; (b) completing expert evaluations based on this graphical structure, utilizing SAM to process the results of expert evaluations and obtain probability information. The DBN graphical structure is derived from the HAZOP results, extracting static nodes, dynamic nodes, and consequence nodes from HAZOP, and determining the potential risk states for each node. The HAZOP results are presented in the form of an accident chain, where the implicit causal relationships represent the risk propagation path. By extracting this risk propagation path and combining it with the direction of risk diffusion, the DBN topology structure for risk diffusion can be constructed. Table 4 summarizes the specific steps and requirements for constructing a risk dynamic evolution analysis model based on the FDBN model construction methodology.

### 3.3. Risk Assessment

The risk assessment is conducted through quantitative analysis of the risk evolution process based on the results from FDBN, encompassing inferential diagnosis, sensitivity analysis, and risk trend analysis. The aim of the risk assessment is to identify key events that could lead to functional anomalies in the onboard hydrogen system and to analyze the underlying causes of these failures. Inferential diagnosis employs the posterior probabilities derived from the FDBN to identify the primary causes of unacceptable consequences. Sensitivity analysis focuses on identifying the main contributors to hazardous events in the onboard hydrogen system based on the ratio of variation (RoV) [70] values. Risk trend analysis synthesizes the results from inferential diagnosis and sensitivity analysis to assess the different degrees of component aging facilitates, subsequent safety analysis, and the establishment of safety constraints. Table 5 presents the specific requirements for each step in the risk assessment process.

(12) $$RoV(X_{i})=\frac{\pi (X_{i})-\theta (X_{i})}{\theta (X_{i})}$$

where $X_{i}$ represents the root node event, $RoV(X_{i})$ is the RoV value of $X_{i}$, $\theta (X_{i})$ is the prior probability of $X_{i}$, and $\pi (X_{i})$ is the posterior probability of $X_{i}$.

### 3.4. Establish Safety Constraints

The results of the risk assessment provide theoretical basis and data support for the designation of safety constraints. Identify the key components that trigger risk events through the risk assessment results and determine the involvement of key components in the risk spreading evolution based on the quantitative analysis results. Based on the component failure stage classified by the trend of component risk state change, reasonable risk prevention and control opinions are formulated. The core idea of formulating safety constraints is to use the model characteristics of FDBN to reduce the initial risk probability, weaken the risk cascade diffusion capability and shorten the risk diffusion time, etc., so as to achieve the reduction in risk diffusion amplitude and weaken the impact of risk propagation.

## 4. Case Analysis

The following analysis is a detailed case study of how the HAZOP-FDBN integration approach is applied to an onboard hydrogen system. The object of analysis is selected as the onboard hydrogen storage system, and the detailed process of HAZOP-based risk factor capture, FDBN dynamic risk assessment model construction, safety assessment, and safety constraint formulation are presented.

### 4.1. Identification of Risk Factors

#### 4.1.1. Overview of Onboard Hydrogen Systems

**Figure 4:**
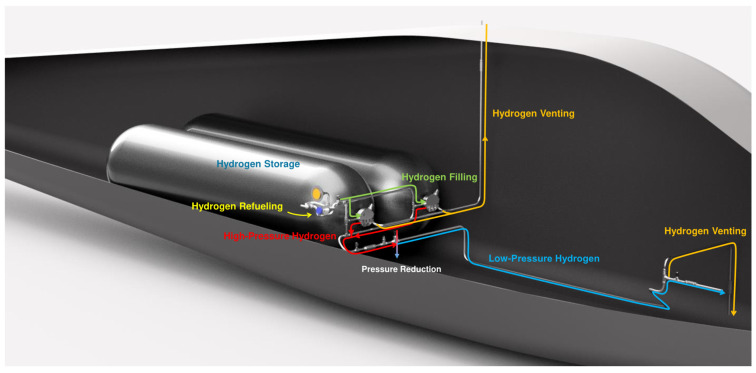
Schematic diagram of the onboard hydrogen system.

The hydrogen system on hydrogen-powered aircraft is primarily responsible for storing high-pressure hydrogen and delivering hydrogen fuel to the power unit. It consists of various valves and interconnected pipelines. The main functions of the onboard hydrogen system include hydrogen refueling, high-pressure hydrogen storage, hydrogen fuel transportation, and hydrogen venting during emergency situations. The analysis in this paper focuses on the hydrogen system of a hydrogen internal combustion engine aircraft, as illustrated in Figure 4.

**Figure 5:**
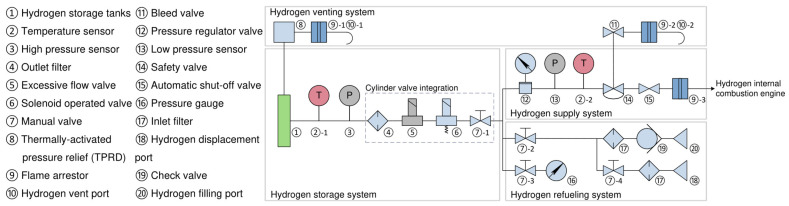
Composition and architecture of the onboard hydrogen system.

The yellow marker indicate hydrogen refueling ports, where hydrogen is loaded and transported via refueling pipelines (marked in green) to the hydrogen storage cylinder assembly for high-pressure storage. During operation, hydrogen delivery begins at the storage cylinder assembly. High-pressure hydrogen (marked in red) undergoes a pressure reduction process to become low-pressure hydrogen (marked in blue) before being supplied to the hydrogen internal combustion engine. Emergency venting routes (marked in orange) are installed near the hydrogen cylinders and the engine to enable rapid hydrogen discharge in critical scenarios. Based on these functions, the hydrogen system can be divided into four subsystems: the hydrogen storage system, the hydrogen refueling system, the hydrogen supply system, and the hydrogen venting system. Each subsystem corresponds to a specific function of the onboard hydrogen system, all achieved through coordinated operation of multiple valves. Figure 5 presents the hydrogen system architecture diagram, visually demonstrating the interconnections between components, while Table 6 details the functional descriptions of each subsystem and the hydrogen supplying process, clarifying the collaborative mechanisms among components.

#### 4.1.2. Node Division

Based on the architecture of the onboard hydrogen system and its operational principles, the subsystems “hydrogen storage system”, “hydrogen refueling system”, “hydrogen supply system”, and “hydrogen venting system”—are designated as the analysis nodes of HAZOP. The correspondence between the node numbers and the subsystems is provided in Table 7.

In this study, node 1 is used as an example to present the analysis process and results of HAZOP.

#### 4.1.3. Clarify the Design Intent

According to Table 6, the hydrogen storage system comprises two units that perform specific functions: hydrogen storage tanks and cylinder valve integration. HAZOP requires a clear understanding of the functions of each component within these units. Table 8 provides a detailed introduction to all components and their functions within these units.

#### 4.1.4. Qualitative Identification Results of Risk Factors

Based on the conclusions outlined above, hydrogen supply pressure, hydrogen supply flow rate, and hydrogen temperature in the tanks are selected as the key process parameters. Taking hydrogen supply pressure as an example, both excessively high or low hydrogen supply pressures can lead to severe system hazards. Notably, excessive hydrogen supply pressure may arise from operational errors during the refueling process and is therefore excluded from the HAZOP of the hydrogen storage system. Consequently, the guideword ‘Too low’ is designated for this parameter. In the example of this study, Functional causes and Component causes were used as the analysis results for the causes of deviations, Functional causes elucidate the direct reasons for process deviations, while component causes describe the specific failure modes of components, serving to refine the functional causes. The results of HAZOP for the onboard hydrogen storage system are presented in Table 9.

### 4.2. Construction of Risk Diffusion Network

Assume that the hydrogen-powered aircraft is to perform 500 missions without maintenance. The flight missions are required to meet the following assumptions:

- It is assumed that the aircraft is in the same condition for each mission, with no consideration given to potential equipment failures due to human factors prior to takeoff.
- It is assumed that environmental conditions for each mission do not include sudden environmental factors like bird strikes and lightning. (According to airworthiness standards, the effect of bird strikes and lightning require dedicated testing, such as bird strike testing and HIRF testing).
- It is assumed that the aircraft performs the same task in each including identical takeoff and landing locations, routes, and task profiles.

#### 4.2.1. Extract Network Nodes

Static nodes, dynamic nodes, and consequence nodes need to determine their status based on the HAZOP results. Based on Table 9, the static nodes are identified as hydrogen supply pressure, hydrogen supply flow rate, hydrogen temperature in tank, and hydrogen pressure in tank, denoted by symbols S1, S2, S3, and S4, respectively. Static nodes are shown in Table 10.

Dynamic nodes are derived from deviation cause analysis. In this study, dynamic nodes are classified into intermediate dynamic nodes and root dynamic nodes. Extract intermediate dynamic nodes and root dynamic nodes from functional reasons and component reasons, respectively. The intermediate dynamic node is sub-ordinate to the root dynamic node; therefore, both types of dynamic nodes exhibit dynamic change characteristics. Dynamic nodes are shown in Table 11.

The consequence nodes are determined based on the consequences of the accident, as shown in Table 12.

#### 4.2.2. Determine Network Connectivity

The connection methodology of the risk diffusion network topology must be determined through risk diffusion paths. This section will detail the process of mapping risk propagation paths via HAZOP result and constructing the risk diffusion network topology based on these identified paths.

1. Risk propagation path

**Figure 6:**
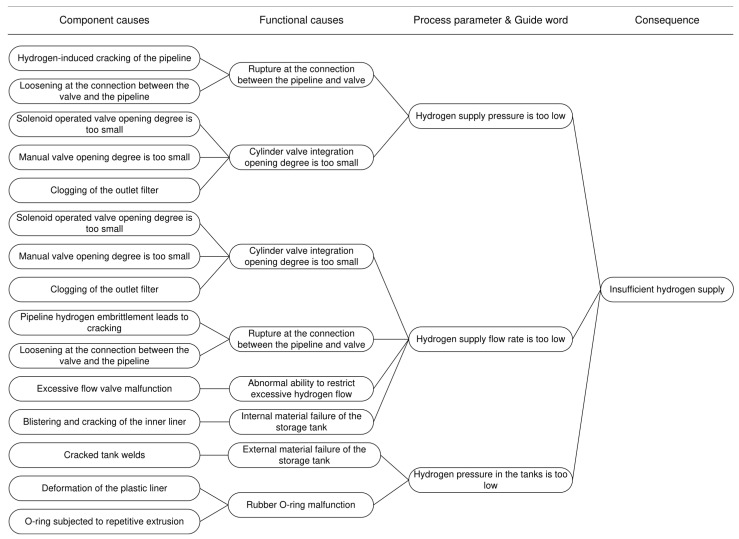
Risk propagation path.

Taking the accident consequence “insufficient hydrogen supply” as an example, this demonstrates the construction process of the risk propagation path. Process parameters and guidewords are directly combined using “is” or “are” (e.g., “Hydrogen supply pressure is too low”). The resulting risk propagation path is illustrated in Figure 6.

2. Risk diffusion network topology

**Figure 7:**
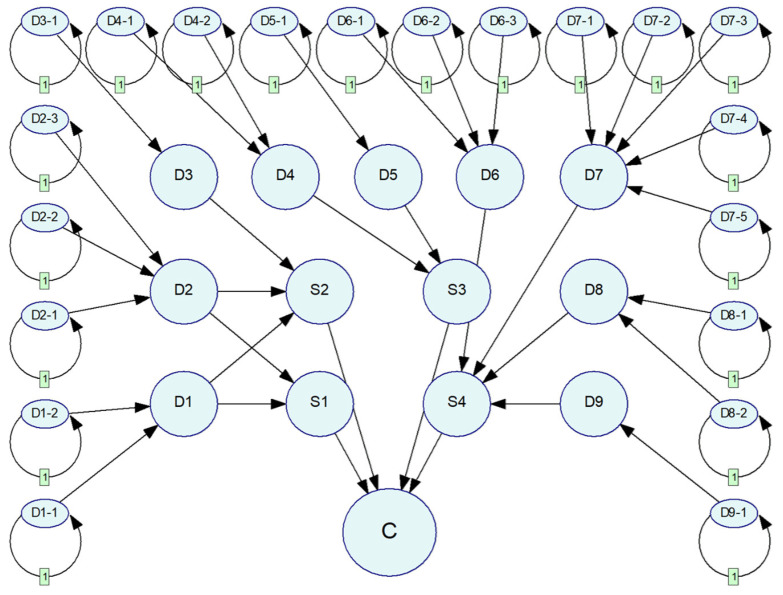
Risk diffusion network topology.

By aligning nodes in the risk propagation path with dynamic nodes, static nodes, and consequence nodes, the risk diffusion network topology is established based on the direction of risk diffusion along the propagation path. The resulting network structure is shown in Figure 7.

#### 4.2.3. Determine Node States

The determination rules for each node’s state vary. This section will provide a detailed elaboration on the state acquisition rules of different nodes.

The risk states of each node are extracted based on the HAZOP results: dynamic node states are extracted from deviation causes; static node states are extracted from guide words; and consequence node states are obtained from accident consequences. According to Table 9 results, each node possesses a normal state along with one or two risk states, referred to as dual-state nodes and three-state nodes, respectively. For dual-state nodes, State 0 and State 1 represent the risk state and normal state, respectively; for three-state nodes, States 0 and 2 denote risk states while State 1 indicates the normal state.

All static node risk states exhibit directional characteristics (e.g., “too high”, “too low”), which should serve as State 0 prompt terms, with State 1 defined as “Normal”. Dual-state nodes in dynamic nodes and consequence nodes lack directionality—State 0 is defined as “True” and State 1 as “False”. Three-state nodes exhibit directional characteristics, using the directional characteristics to define States 0 and 2, with State 1 designated as “Normal”.

The static node state definitions are shown in Table 10, dynamic node state results in Table 11, and consequence node state definitions in Table 12.

#### 4.2.4. Obtain Probability Information

The expert evaluates the likelihood of risk state occurrence for each node based on the DBN graphical structure and transforms the expert evaluation results into fuzzy probabilities according to the transformation method introduced in Section 2.3, thus obtaining the probabilistic information required for constructing the FDBN model. The probabilistic information required includes five types of probabilities: the prior probabilities of root dynamic nodes, the state transfer probability of root dynamic nodes, the conditional probabilities of intermediate dynamic nodes, the conditional probabilities of static nodes, and the conditional probabilities of consequence nodes.

The reliability level of the expert evaluation depends on the expert opinion weights, thus, the expert scoring criteria shown in Table 2 are used to calculate these weights. The information on the experts involved in this study was organized, and the weight values of each expert were calculated, as shown in Table 13.

Based on the results of the expert evaluations, the rules for obtaining each probability are presented below.

1. Prior probabilities of root dynamic nodes

**Figure 8:**
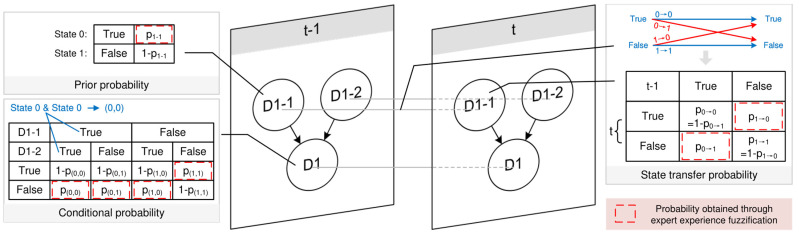
Probability acquisition rules.

The priori probability of root dynamic nodes is the probability of the root dynamic node occurring at the initial time slice. For dynamic nodes, determining the probabilities of risk statuses requires expert experience. As illustrated in Figure 8, taking D1-1 as an example, the probability P_1-1_ of State 0 (“True”) is first determined. The probability of State 1 (“False”) is then calculated based on the principle that the sum of probabilities equals 1. The prior probabilities of each node are shown in Table 14.

2. State transfer probabilities of root dynamic nodes

The expert evaluates the transfer probabilities for the state of the root dynamic node, and the notation 0→1 is used to indicate the transition from node State 0 to node State 1.

As shown in Figure 8, taking node D1-1 from time slice t − 1 to t as an example, explain the state transition probability evaluation rules. As shown in Figure 8, all possible state transitions for the node are: 0→0, 0→1, 1→0, and 1→1. Among these are the following:

- 0→1 and 1→0 represent state changes, and their transition probabilities P_0→1_ and P_1→0_ are obtained through expert experience.
- 0→0 and 1→1 indicate the maintenance of the original state (no change). These probabilities are calculated based on the principle that probabilities sum to 1, i.e., P_0→0_ = 1 − P_0→1_, P_1→1_ = 1 − P_1→0_.

Table 15 presents the calculated state transition probability results for node D1-1.

3. Conditional probabilities of intermediate dynamic nodes

The parent node of the intermediate dynamic nodes is the root dynamic node, so the conditional probability of the intermediate dynamic node is the probability of the occurrence of the intermediate dynamic node’s risk state given the different state combinations of the root dynamic node.

As shown in Figure 8, taking node D1 and its parent nodes D1-1 and D1-2 as examples, explain the expert evaluation rules for obtaining the conditional probability of intermediate dynamic nodes. Using the coordinates (0,0) to denote that both parent nodes D1-1 and D1-2 are in State 0, all possible state combinations are (0,0), (0,1), (1,0), and (1,1).

- Combinations (0,0), (0,1), and (1,0), which involve at least one parent node in a risk state (State 0), thus determines the probability of State 1 (“False”) for node D1 through expert experience.
- Combination (1,1), where no parent node is in a risk state, thus determines the probability of State 0 (“True”) for node D1 though expert experience.

All remaining probabilities are calculated based on the principle that probabilities come to 1.

The calculated conditional probability results for node D1 are summarized in Table 16.

4. Conditional probabilities of static nodes and consequence nodes

The parent node of a static node is an intermediate dynamic node, and the parent node of a consequence dynamic node is a static node. The conditional probability of static node and consequence node acquisition method is the same as the intermediate dynamic node conditional probability acquisition method and will not be repeated here.

**Figure 9:**
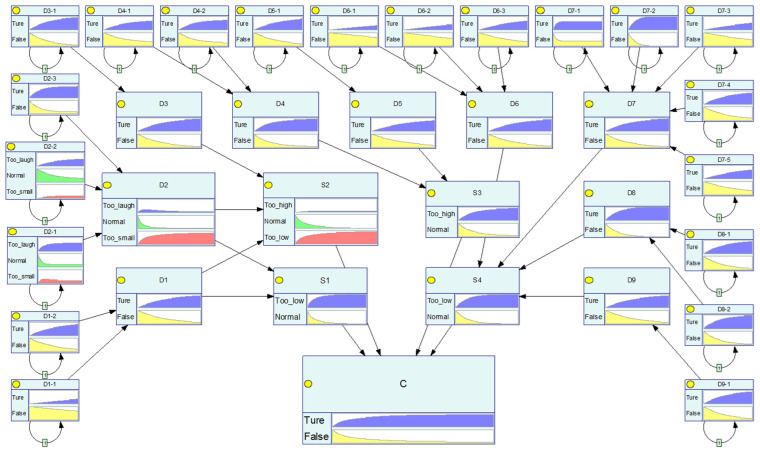
Computation results of the FDBN model.

After calculating all probability information using the aforementioned method, the risk diffusion network structure is integrated with the probability data. Using the FDBN model to compute the risk probability variations for each node state. The calculation results are illustrated in Figure 9.

### 4.3. Dynamic Assessment of Risk Evolution

Based on the results of the FDBN calculation, the risk assessment of the onboard hydrogen storage system will be conducted through inferential diagnosis, sensitivity analysis, and risk trend analysis, Provide basis for the establishment of safety constraints. Based on empirical judgment, the State 0 of node D2-1 (solenoid operated valve opening degree is too large) and the State 0 of node D2-2 (manual valve opening degree is too large) are not expected to lead to the event of “insufficient hydrogen supply”. Therefore, these two states are not considered in the subsequent risk assessment.

#### 4.3.1. Inferential Diagnosis

Inferential diagnosis entails initially establishing the evidence at the consequence node and subsequently calculating the posterior probabilities of the other nodes through the reverse inference capability of the DBN model. This process aims to identify the most probable event of equipment or component failure that resulted in the observed consequence [71,72]. Inferential diagnosis provides a better estimate of the risk state by calculating the posterior probabilities of each node at different time slices [37]. In this study, the probability of node C being in State 0 is set to 1, indicating the occurrence of the “insufficient hydrogen supply” event. Calculate the posterior probabilities of each dynamic node using the backward inference capability of the DBN model, as shown in Table 15 (the tenth column). The posterior probabilities of all root dynamic nodes are sorted from highest to lowest as follows:

D2-2 State 2 > D2-1 State 2 > D2-3 > D7-2 > D1-2 > D7-1 > D8-2 > D8-1 > D3-1 > D4-1 > D7-5 > D7-4 > D6-3 > D9-1 > D5-1 > D1-1 > D6-1 > D4-2 > D6-2 > D7-3

The posterior probabilities of node D2-2 State 2, D2-1 State 2, and D2-3, are 5.11 × 10^−1^ (0.5106), 3.44 × 10^−1^ (0.3442), and 1.31 × 10^−1^ (0.1306), respectively. These values are significantly higher than those of other nodes, indicating that these events are the primary causes of “insufficient hydrogen supply”. Consequently, these issues should be prioritized in aircraft maintenance to mitigate their failure probability.

**Figure 10:**
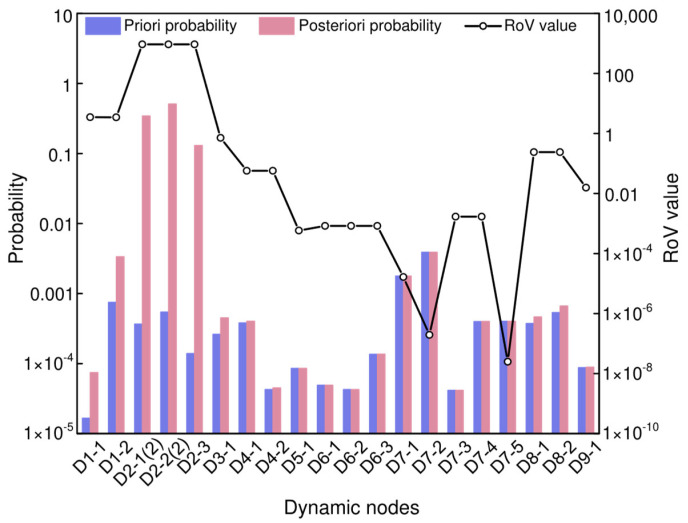
Comparison of the priori and posteriori probabilities with ROV values for dynamic nodes.

The difference between the priori and posterior probabilities indicates the extent of change in the failure probability of the nodes. Figure 10 visually compares the priori and posterior probabilities of root dynamic nodes. From the figure, it can be observed that the difference between the prior and posterior probabilities for node D1-2, node D2-1 State 2, node D2-2 State 2, and node D2-3 are significantly higher compared to other nodes. Therefore, “loosening at the connection between the valve and the pipeline”, “solenoid operated valve opening degree is too small”, “manual valve opening degree is too small”, and “clogging of the outlet filter” are identified as the most probable causes of “insufficient hydrogen supply”.

Summary: “solenoid operated valve opening degree is too small”, “manual valve opening degree is too small”, and “clogging of the outlet filter” may be the primary causes of “inadequate hydrogen supply”, which should be given high priority in aircraft maintenance work. The “loosening at the connection between the valve and the pipeline” may significantly contribute to inadequate hydrogen supply, and aircraft maintenance personnel should monitor its dynamic changes and promptly replace any faulty equipment and parts.

#### 4.3.2. Sensitivity Analysis

Sensitivity analysis can determine the sensitivity of risk events to dynamic root nodes. The RoV value reflects the contribution of the root dynamic nodes to the consequence node and identifies the root dynamic node within the model that exerts the greatest influence on the consequence node [73,74]. According to the information in Table 15 and Figure 10, the RoV values in descending order are as follows:

D2-3 > D2-2 Status 2 > D2-1 Status 2 > D1-1 > D1-2 > D3-1 > D8-2 > D8-1 > D4-2 > D4-1 > D9-1 > D7-4 > D7-3 > D6-1 > D6-2 > D6-3 > D5-1 > D7-1 > D7-2 > D7-5

Among these, the RoV values for nodes D2-3, D2-2 Status 2, and D2-1 Status 2 are 934.3831, 934.0998, and 933.8836, respectively, which are significantly higher than those of other nodes. Therefore, “clogging of the outlet filter”, “manual valve opening degree is too small”, and “solenoid operated valve opening degree is too small” are the primary contributing events to the consequence of “insufficient hydrogen supply”.

#### 4.3.3. Risk Trend Analysis

As the number of missions performed by the aircraft increases, the likelihood of failure in the hydrogen storage system—whether localized or systemic—significantly rises due to component failures. For events requiring attention, it is essential to identify the various aging stages through risk trend analysis. This approach enables the formulation of safety constraints based on the impact of different aging levels on system safety. Section 4.3.1 and Section 4.3.2 indicate that the primary nodes contributing to the consequence of “insufficient hydrogen supply” are node D2-1 State 2, node D2-2 State 2, node D2-3, and node D1-2. The following is an example of node D2-1 State 2, which explains the different stages of solenoid operated valve aging.

**Figure 11:**
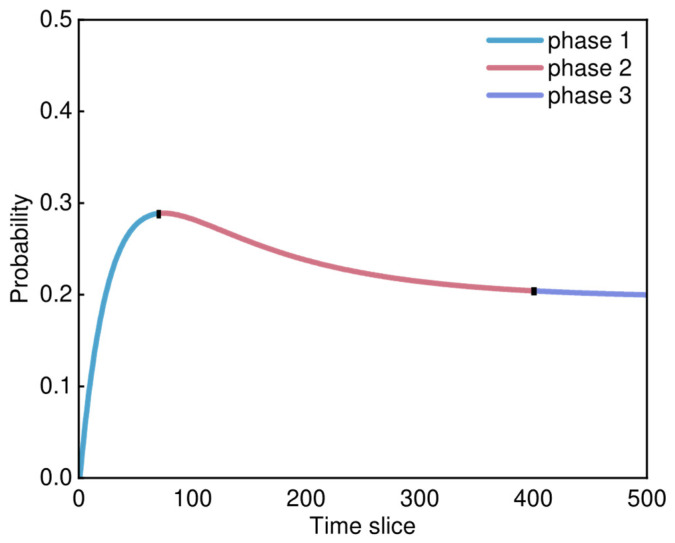
Risk trend of node D2-1 State 2.

Figure 11 shows the risk trend curve of node D2-1 State 2, this curve exhibits three phases of trend changes, namely phase 1, phase 2, and phase 3, as shown in the figure.

In phase 1, the risk trend is in an upward state. This indicates that the aircraft’s execution of flight missions requires the solenoid operated valve to repeatedly open and close, leading to the aging of the valve. At this phase, the valve aging is in the initial stage, and the electronic components within the solenoid operated valve experience a decline in their ability to recognize the valve opening degree. As a result, the valve may become slightly loose, causing excessive or insufficient opening, with this error gradually increasing over time.

In phase 2, the risk trend starts to decline, indicating that with further valve aging, the electronic components have become significantly deteriorated, and the valve has become significantly loose, making it difficult to withstand high-pressure hydrogen gas.

In phase 3, the risk trend of the valve opening too small declines and converges to a lower value, which may be due to corrosion, deformation, and other factors. The valve opening resistance increases or there is a loss of opening capability, and there remains a certain probability of the valve opening being too small.

### 4.4. Case Example of Establishing Safety Constraints

Taking node D2-1 as an example, this section demonstrates how to derive risk prevention and control measures based on risk assessment results. The following will analyze from three aspects: reducing the initial probability value of risk states, modifying the transition probabilities to decrease the likelihood of normal states transitioning to risk states, and shortening the time window length, to derive reasonable preventive measures.

1. Reducing the initial probability value of risk states

Reducing the inherent probability of solenoid operated valves can decrease the likelihood of failure over the same aging period. Reducing the initial probability means that the vertical intercept of the risk curve decreases, causing the entire risk curve to shift downward. This necessitates identifying critical stages in the valve’s life cycle where probabilities can be minimized and establishing appropriate safety constraints at each stage.

- During the design phase, adopting stricter design standards can define requirements that reduce the inherent failure probability. It is recommended to refer to the SAE ARP 4761A standard by adopting a dual solenoid coil redundancy design and providing independent power supply for the redundant system, while establishing a fault switching time tolerance range. Additionally, the valve materials can be upgraded to those with better adaptability in high-pressure hydrogen environments. The solenoid operated valve should also follow the RTCA DO-160G standard to design relevant environmental adaptability tests, evaluating its performance in airborne environments, and implement improvements to enhance its compatibility with onboard operational conditions.
- During the maintenance phase, enforcing rigorous inspection protocols maximizes fault reduction. For the vulnerable soft seals of the valve, implement a dual-control replacement strategy based on ‘operating hours + calendar lifespan’ (replacement is required if either upper limit is reached). Additionally, to mitigate failures due to improper installation, implementing measures such as enhanced worker training and improved installation guidelines is essential.
- Adjusting transition probabilities to minimize the likelihood of normal states evolving into risk states

Solenoid operated valves will experience gradual aging with increased usage, resulting in a heightened likelihood of malfunction. Therefore, reducing the aging speed of these valves can effectively decrease their failure probability.

- Solenoid operated valves installed in various aircraft locations must meet specific design requirements to ensure safety. Selecting valves that align better with the safety standards of their installation sites can help mitigate aging.
- In airborne environments, solenoid operated valves are subject to vibrations, which may significantly accelerate their aging. Consequently, modifying the installation position or incorporating protective devices can reduce vibrational impacts, thereby slowing the aging speed. The vibration response spectrum peaks can be reduced by installing shock-absorbing devices such as dampers. Additionally, the control logic can be optimized to lower the valve actuation frequency during non-critical phases (e.g., the cruise phase).
- Shortening the time window length

Reducing the aging time of solenoid operated valves is essential for keeping their failure probability within an acceptable range. This can be achieved by decreasing maintenance intervals or implementing regular valve replacements.

Due to space constraints, this section only employs solenoid operated valve as a case study to illustrate the process of deriving safety constraints. For other critical components identified from the risk assessment in Section 4.3, appropriate safety constraints can also be established by referring to the analytical framework presented herein.

## 5. Discussion

This study conducted inference diagnosis, sensitivity analysis, and risk trend analysis based on the FDBN model. The inferential diagnosis reasoning identified key causes contributing to “insufficient hydrogen supply”, including “manual valve opening degree is too small”, “solenoid operated valve opening degree is too small”, “clogging of the outlet filter”, and “loosening at the connection between the valve and the pipeline”. Each of these causes has a substantial likelihood of causing hydrogen leakage incidents. Sensitivity analysis indicated that “clogging of the outlet filter”, “manual valve opening degree is too small”, and “solenoid operated valve opening degree is too small” are the primary contributing events to the consequence of “insufficient hydrogen supply”. Based on the results of both inference diagnosis and sensitivity analysis, it is determined that the primary causes of “insufficient hydrogen supply” are “manual valve opening degree is too small”, “solenoid operated valve opening degree is too small”, “clogging of the outlet filter”, and “looseness at the connection between the pipeline and the valve”. Therefore, the integration of HAZOP and FDBN can capture quantitative risk factors in risk propagation. By leveraging HAZOP’s risk identification capabilities and FDBN’s uncertainty reasoning, a comprehensive risk assessment for onboard hydrogen systems can be achieved.

Risk trend analysis focuses on these main causes, tracing the evolution of failure processes to facilitate the formulation of risk prevention and control measures, and imposing safety constraints on onboard hydrogen systems. This study selected “opening degree of the solenoid operated valve is too small” for risk trend analysis, systematically analyzing the three trend phases of node D2-1 in State 0: (phase 1) The solenoid operated valve undergoes repeated opening and closing in a high-pressure environment, resulting in the initial loosening of mechanical connections; (phase 2) the solenoid operated valve shows significant aging, with failures in electronic components, leading to a decline in the valve’s ability to control hydrogen; (phase 3) the solenoid operated valve is severely aged, with nearly complete loss of functionality in both electronic components and mechanical connections, resulting in a complete loss of operational capability of the solenoid operated valve.

Based on the risk assessment results, a safety constraint is proposed, illustrated by the scenario of “solenoid operated valve opening degree is too small”. This constraint targets three key aspects: reducing the initial probability value of risk states, adjusting transition probabilities to minimize the likelihood of normal states evolving into risk states, and shortening the time window length. Five measures are presented in Section 4.4 covering essential safety requirements throughout the design, installation, and maintenance of solenoid operated valves. An iterative risk assessment process can promote the development of a comprehensive risk prevention and control plan, with the method introduced in Section 4.4 serving as the primary framework. Defining safety constraints as limiting nodes to update the FDBN model. Following the computational analysis of the updated FDBN, more detailed safety constraints will be developed through further risk assessments. This iterative process will persist until the result meets the established safety requirements, at which point the iteration will conclude.

## 6. Conclusions

To address the challenge of quantitatively modeling the risk diffusion process in onboard hydrogen systems without empirical data and to mitigate the reliance of existing quantitative risk assessment methods on complete and accurate data sources, this study proposes a HAZOP-FDBN-based risk assessment method for the evaluation of risk evolution within the system. The method employs HAZOP and FDBN as analytical tools to construct a risk diffusion network for risk factors, enabling quantitative assessment of dynamic risks. Specifically, HAZOP is used to identify risk elements by determining process deviations, causes of deviations, and accident consequences, thereby comprehensively obtaining risk factors in the onboard hydrogen system. The risk propagation relationships mapped by HAZOP are utilized to establish a network topology structure based on risk diffusion directions. Collecting expert evaluations results and then processed through fuzzification and defuzzification to derive fuzzy failure probabilities. By integrating the risk diffusion topology with these fuzzy failure probabilities, an FDBN-based risk diffusion assessment model is developed.

Using the computational results from this model, the method accomplishes risk assessment through inference diagnosis, sensitivity analysis, and risk trend analysis. Finally, safety constraint recommendations are provided based on the risk assessment outcomes. Inferential diagnosis identifies that “solenoid operated valve opening degree is too small”, “manual valve opening degree is too small”, “clogging of the outlet filter”, and “loosening at the connection between the valve and the pipeline” are key causes of “insufficient hydrogen supply”; sensitivity analysis determines that “solenoid operated valve opening degree is too small”, “manual valve opening degree is too small”, and “clogging of the outlet filter” are the main contributing events to “insufficient hydrogen supply”; risk trend analysis assesses the aging degree of the solenoid operated valve by evaluating the different stages of the risk trend; analyze the key components identified in the risk assessment results that contribute to risks to develop risk prevention and control measures, which will serve as safety constraints for the system.

However, there are still the following issues for this study that need to be addressed in future research:

1. The transition probability of nodes in this study is set to a fixed value, which makes it difficult to characterize the fluctuation of state transition probability across time intervals. This is a practical approach for the early stages of lacking operational monitoring data, but when hydrogen-powered aircraft is put into operation and sufficient monitoring data are accumulated, the transition probability of nodes should be set to more realistic variable values, and the risk analysis results will be more refined.
2. When practical application data are accumulated, the risk assessment model must be updated and adjusted in real-time to adapt to evolving system operating conditions. Specifically, the observed data from the real-world can be incorporated into the corresponding nodes of the FDBN model as evidence. These evidence values serve as the basis for aligning the model with actual operational scenarios, thereby enabling the iterative refinement of the quantitative risk assessment model.

## Figures and Tables

**Table 1:** The fuzzy sets corresponding to the linguistic terms.

Linguistic Value	Fuzzy Set			
	a	b	c	d
VL	0	0	0.1	0.2
L	0.1	0.2	0.2	0.3
ML	0.2	0.3	0.4	0.5
M	0.4	0.5	0.5	0.6
MH	0.5	0.6	0.7	0.8
H	0.7	0.8	0.8	0.9
VH	0.8	0.9	1	1

**Table 2:** Weighting criteria and weight score of experts.

Constitution	Classification	Weight Score
Professional title	Technical director	10
	Technical consultant	8
	Engineer	6
Research duration (years)	≥20	10
	15~19	8
	10~14	6
	6~9	4
Age (years old)	≥50	10
	40~49	8
	30~39	6

**Table 3:** Steps and requirements for HAZOP qualitative identification of risk factors.

Step	Description
Division of nodes	According to the design intent of the onboard hydrogen system, the system is decomposed into subsystems with different functions, which are considered as nodes for HAZOP.
Clarify the design intent	Clearly define the design intent of the subsystems and all their components, including the hydrogen supply process, subsystem functions, and the functions of each component.
Determine process parameters	The operational integrity of the system requires that critical parameters be maintained within specified safety thresholds, and critical design parameters whose deviations may trigger hazardous system events need to be identified.
Analyze deviations	The core of HAZOP lies in analyzing deviations, which involves four main steps: (a) Analysis of potential deviations in process parameters System abnormal operating states manifest through deviations in state parameters, necessitating screening of potential deviations that may induce functional anomalies. (b) Identification of deviation guide words Process parameters may deviate in distinct directions. Guide words are employed to intuitively express deviation orientations, establishing standardized terminology to define analytical objectives. (c) Tracing root causes of deviations Process parameter deviations originate from abnormal operations of system components or functional units. By correlating with system architecture, this traces risk propagation paths from fault sources to measurable parameter deviations, analyzing causation mechanisms. (d) Assessment of deviation consequences Process deviations propagate to system-level manifestations, demonstrating hazardous events caused by abnormal operations. This clarifies cascading impact progression and identifies system-level hazardous consequences induced by deviations.
Iteration of the analysis process	Risk assessment shall be anchored in safety-driven design requirements allocation, with evaluation results requiring completeness validation of analysis nodes and demonstration of requirement traceability to fulfill verification objectives.
Qualitative identification results of risk factors	Organize process parameters, deviation guide words, root causes, and consequences in a table format to form the final risk factor identification results.

**Table 4:** Steps and requirements for FDBN construction of risk diffusion network model.

Step	Description
Extract network nodes	Extract the nodes of the DBN model from the results of the HAZOP, designating the causes of deviations as dynamic nodes, the process parameters as static nodes, and the accident consequences as consequence nodes.
Determine network connectivity	HAZOP is based on the risk propagation relationships involving root causes, process deviations, and consequences. According to the risk factors identification results table, it is possible to determine the connections between dynamic nodes, static nodes, and consequence nodes. Use risk propagation paths to represent these relationships and map them into the topological structure of the risk diffusion network.
Determine node states	Each node has normal and risk states. The risk states of dynamic nodes are determined by the causes of process parameter deviations. The risk states of static nodes are determined by deviation guide words. The risk states of consequence nodes are determined by the consequences of process deviations leading to accidents.
Obtain probability information	Probability information is obtained as probabilistic information through fuzzy processing expert experience, which is divided into five steps in total: (a) Expert evaluation The expert evaluates the probability of all risk factors according to the system architecture and collects the results of the expert evaluation. (b) Quantification of fuzzy results Based on steps 1 to 6 of SAM introduced in Section 2.3, the expert evaluation results are integrated into overall fuzzy numbers. (c) Calculate FFP Based on Steps 7 and 8 of the SAM introduced in Section 2.3, the overall fuzzy numbers are defuzzified to obtain the fuzzy failure probabilities. The node states that need to determine the risk probability through defuzzified expert opinions are more flexible and need to be judged based on the actual system architecture constructed by DBN. Usually, the root node needs to determine the a priori probability, and if the root node is a dynamic node, it also needs to determine the state transfer probability of the risk state; the intermediate node and the leaf node need to determine the conditional probability of the risk state.
Risk dynamic evolution assessment model	Integrate the topology network structure and risk probability information of DBN to develop an FDBN model as a risk dynamic evolution assessment model.

**Table 5:** Steps and requirements for risk assessment.

Step	Description
Inferential diagnosis	The probability of occurrence of the risk state in the BN for the initial time slice is set to 1. The posteriori probability of each root node is obtained by updating the model using DBN backward inference. Events represented by nodes with higher posterior probabilities are identified as the primary events that contribute to the occurrence of hazardous incidents.
Sensitivity analysis	Based on prior probabilities and posterior probabilities, the RoV values are calculated using Equation (12). Nodes with higher RoV values are identified as the main dependent nodes of consequence nodes, thereby establishing the events represented by these nodes as significant contributors to the occurrence of hazardous incidents.
Risk trend analysis	Select the primary components identified through inferential diagnosis and sensitivity analysis as contributing to the risks associated with the onboard hydrogen system. Based on the results of the FDBN calculations, analyzing the failure probability change curves to identify the different stages of components from normal to failure.

**Table 6:** Subsystems and functions of the onboard hydrogen system.

Subsystem	Supplying Process	System Functions
Hydrogen storage system	$①⇄②\text{-}1⇄③⇄\mathrm{cylinder}\;\mathrm{valve}\;\mathrm{integration}\;(④⇄⑤⇄⑥⇄⑦\text{-}1)⇄H_{2}\;①\to H_{2}\to \mathrm{hydrogen}\;\mathrm{venting}\;\mathrm{system}\;(⑧)$	Storage of high-pressure hydrogen.
		Regulation of high-pressure hydrogen supply and hydrogen flow.
		Management of hydrogen discharge from storage cylinders.
Hydrogen refueling system	$H_{2}\to ⑳\to ⑲\to ⑰\to ⑦\text{-}2\to \mathrm{hydrogen}\;\mathrm{storage}\;\mathrm{system}\;(⑦\text{-}1)$	Ensure rapid and stable hydrogen refilling.
	$H_{2}\to ⑱\to ⑰\to ⑦\text{-}4\to ⑦\text{-}2\to \mathrm{hydrogen}\;\mathrm{storage}\;\mathrm{system}\;(⑦\text{-}1)$	
Hydrogen supply system	$\mathrm{Hydrogen}\;\mathrm{storage}\;\mathrm{system}\;(⑦\text{-}1){\to H}_{2}\to ⑫\to ⑬\to ②\text{-}2\to ⑭\to ⑮\to ⑨\text{-}3\;\to \mathrm{hydrogen}\;\mathrm{internal}\;\mathrm{combustion}\;\mathrm{engine}$	Reduce the pressure of high-pressure hydrogen gas and ensure a stable supply to the hydrogen internal combustion engine.
Hydrogen venting system	$\mathrm{Hydrogen}\;\mathrm{storage}\;\mathrm{system}\;(①){\to H}_{2}\to ⑧\to ⑨\text{-}1\to ⑩\text{-}1$	Rapidly and safely vent hydrogen in specific situations.
	${\mathrm{Hydrogen}\;\mathrm{supply}\;\mathrm{system}\;(⑭)\to H}_{2}\to ⑪\to ⑨\text{-}2\to ⑩\text{-}2$	

**Table 7:** Correspondence of HAZOP node numbers to subsystems.

Node Number	Subsystem
Node 1	Hydrogen storage system
Node 2	Hydrogen refueling system
Node 3	Hydrogen supply system
Node 4	Hydrogen venting system

**Table 8:** Composition and functions of the integration unit in hydrogen storage systems.

Unit	Component	Function
Hydrogen storage tank	Type IV hydrogen storage cylinders	Store high-pressure hydrogen gas.
	Temperature sensor	Measure the hydrogen temperature in storage cylinders.
	High pressure sensor	Measure the hydrogen pressure in storage cylinders.
Cylinder valve integration	Outlet filter	Filter impurities from the hydrogen gas.
	Excessive flow valve	Automatic cut-off of the gas flow when the hydrogen flow rate seriously exceeds the usage limit.
	Solenoid operated valve	Adjust hydrogen flow based on signals.
		Control the start/stop of the hydrogen supply based on signals.
	Manual valve	The operator manually controls the hydrogen supply.

**Table 9:** HAZOP results of the onboard hydrogen storage system.

Process Parameter	Guide Word	Cause		Consequence	Safety Measures
		Functional Causes	Component Causes		
Hydrogen supply pressure	Too low	Rupture at the connection between the pipeline and valve	Pipeline hydrogen embrittlement leads to cracking	Hydrogen leakage Insufficient hydrogen supply Unstable combustion in hydrogen internal combustion engines	Regular maintenance of the pipeline Install hydrogen leakage detection device Timely removal of leaked hydrogen gas
			Loosening at the connection between the valve and the pipeline	Hydrogen leakage Insufficient hydrogen supply Unstable combustion in hydrogen internal combustion engines	Install hydrogen leakage detection device Timely removal of leaked hydrogen gas Regularly check the tightness of the pipeline -valve connection
		Cylinder valve integration opening degree is too small	Solenoid operated valve opening degree is too small	Insufficient hydrogen supply Unstable combustion in hydrogen internal combustion engines	Regular maintenance of the solenoid operated valve
			Manual valve opening degree is too small	Insufficient hydrogen supply Unstable combustion in hydrogen internal combustion engines	Regular maintenance of the manual valve
			Clogging of the outlet filter	Insufficient hydrogen supply Unstable combustion in hydrogen internal combustion engines	Periodic replacement of the outlet filter
Hydrogen supply flow rate	Too high	Cylinder valve integration opening degree is too large	Solenoid operated valve opening degree is too large	Pre-ignition Backfire	Regular maintenance of the solenoid operated valve
			Manual valve opening degree is too large	Pre-ignition Backfire	Regular maintenance of the manual valve
		Abnormal ability to restrict excessive hydrogen flow	Excessive flow valve malfunction	Unstable combustion in hydrogen internal combustion engines Pre-ignition Backfire	Regular maintenance of the excessive hydrogen flow
	Too low	Cylinder valve integration opening degree is too small	Solenoid operated valve opening degree is too small	Insufficient hydrogen supply Unstable combustion in hydrogen internal combustion engines	Regular maintenance of the solenoid operated valve
			Manual valve opening degree is too small	Insufficient hydrogen supply Unstable combustion in hydrogen internal combustion engines	Regular maintenance of the manual valve
			Clogging of the outlet filter	Insufficient hydrogen supply Unstable combustion in hydrogen internal combustion engines	Periodic replacement of the outlet filter
		Rupture at the connection between the pipeline and valve	Pipeline hydrogen embrittlement leads to cracking	Hydrogen leakage Insufficient hydrogen supply Unstable combustion in hydrogen internal combustion engines	Regular maintenance of the pipeline Install hydrogen leakage detection device Timely removal of leaked hydrogen gas
			Loosening at the connection between the valve and the pipeline	Hydrogen leakage Insufficient hydrogen supply Unstable combustion in hydrogen internal combustion engines	Install hydrogen leakage detection device Timely removal of leaked hydrogen gas Regularly check the tightness of the pipeline -valve connection
		Abnormal ability to restrict excessive hydrogen flow	Excessive flow valve malfunction	Unstable combustion in hydrogen internal combustion engines Pre-ignition Backfire	Regular maintenance of the excessive hydrogen flow
Hydrogen temperature in the tanks	Too high	Hydrogen emission delay	TPRD fails to fully open	Increased burden on the thermal management system	Regular maintenance of the TPRD
			Excessive buildup of deposits in the emptying pipeline or hydrogen vent port	Increased burden on the thermal management system	Regular cleaning of the emptying pipeline and hydrogen vent port
		Lack of accurate temperature feedback	Temperature sensor malfunction	Increased burden on the thermal management system	Regular replacement of the temperature sensor
Hydrogen pressure in the tanks	Too low	Internal material failure of the storage tank	Blistering and cracking of the inner liner	Hydrogen leakage Insufficient hydrogen supply Reduction in hydrogen storage efficiency	Regular replacement of the hydrogen storage tanks
			Bulging and collapse of the inner liner	Reduction in hydrogen storage efficiency	Regular replacement of the hydrogen storage tanks
			Fatigue damage to the inner liner	Reduction in hydrogen storage efficiency	Regular replacement of the hydrogen storage tanks
		External material failure of the storage tank	Repeated cyclic stamping of the tank body	Reduction in hydrogen storage efficiency	Regular replacement of the hydrogen storage tanks
			Scratches on the tank surface	Promote the formation of surface cracks on the tank body	Regular replacement of the hydrogen storage tanks
			Hydrogen embrittlement cracking of the tank body	Hydrogen leakage Insufficient hydrogen supply	Regular replacement of the hydrogen storage tanks
			Cracked tank welds	Hydrogen leakage Insufficient hydrogen supply	Regular replacement of the hydrogen storage tanks
			Residual stress exists in the tank	Reduction in hydrogen storage efficiency Promote the formation of surface cracks on the tank body	Regular replacement of the hydrogen storage tanks
		Rubber O-ring malfunction	Deformation of the plastic liner	Hydrogen leakage Insufficient hydrogen supply	Regular replacement of O-rings
			O-ring subjected to repetitive extrusion	Hydrogen leakage Insufficient hydrogen supply	Regular replacement of O-rings
		Lack of accurate pressure feedback	High pressure sensor malfunction	Unstable combustion in hydrogen internal combustion engines	Regular replacement of the high-pressure sensor

**Table 10:** Static nodes of the DBN model.

Node Symbol	Static Node Name	State
S1	Hydrogen supply pressure	0: Too low 1: Normal
S2	Hydrogen supply flow rate	0: Too high 1: Normal 2: Too low
S3	Hydrogen temperature in tank	0: Too high 1: Normal
S4	Hydrogen pressure in tank	0: Too low 1: Normal

**Table 11:** Dynamic nodes of the DBN model.

Root Dynamic Nodes			Intermediate Dynamic Nodes		
Node Symbol	Node Name	State	Node Symbol	Node Name	State
D1	Rupture at the connection between the pipeline and valve	0: True 1: False	D1-1	Pipeline hydrogen embrittlement leads to cracking	0: True 1: False
			D1-2	Loosening at the connection between the valve and the pipeline	0: True 1: False
D2	Cylinder valve integration opening degree	0: Too large 1 Normal 2: Too small	D2-1	Solenoid operated valve opening degree	0: Too large 1: Normal 2: Too small
			D2-2	Manual valve opening degree	0: Too large 1: Normal 2: Too small
			D2-3	Clogging of the outlet filter	0: True 1: False
D3	Abnormal ability to restrict excessive hydrogen flow	0: True 1: False	D3-1	Excessive flow valve malfunction	0: True 1: False
D4	Hydrogen emission delay	0: True 1: False	D4-1	TPRD fails to fully open	0: True 1: False
			D4-2	Excessive buildup of deposits in the emptying pipeline or hydrogen vent port	0: True 1: False
D5	Lack of accurate temperature feedback	0: True 1: False	D5-1	Temperature sensor malfunction	0: True 1: False
D6	Internal material failure of the storage tank	0: True 1: False	D6-1	Blistering and cracking of the inner liner	0: True 1: False
			D6-2	Bulging and collapse of the inner liner	0: True 1: False
			D6-3	Fatigue damage to the inner liner	0: True 1: False
D7	External material failure of the storage tank	0: True 1: False	D7-1	Repeated cyclic stamping of the tank body	0: True 1: False
			D7-2	Scratches on the tank surface	0: True 1: False
			D7-3	Hydrogen embrittlement cracking the tank body	0: True 1: False
			D7-4	Cracked tank welds	0: True 1: False
			D7-5	Residual stress exists in the tank	0: True 1: False
D8	Rubber O-ring malfunction	0: True 1: False	D8-1	Deformation of the plastic liner	0: True 1: False
			D8-2	O-ring subjected to repetitive extrusion	0: True 1: False
D9	Lack of accurate pressure feedback	0: True 1: False	D9-1	High pressure sensor malfunction	0: True 1: False

**Table 12:** Consequence nodes of the DBN model.

Node Symbol	Node Name	State
C1	Hydrogen leakage	0: True 1: False
C2	Insufficient hydrogen supply	0: True 1: False
C3	Unstable combustion in hydrogen internal combustion engines	0: True
C4	Pre-ignition	1: False
C5	Backfire	0: True 1: False
C6	Increased burden on the thermal management system	0: True 1: False
C7	Reduction in hydrogen storage efficiency	0: True 1: False
C8	Promote the formation of surface cracks on the tank body	0: True 1: False

**Table 13:** Expert information and weight expert information and weight values.

Expert	Professional Title	Research Duration (Years)	Age (Years Old)	Weight Score	Weight Value
1	Technical consultant	15–19	≥50	24	0.218
2	Technical director	≥20	40–49	26	0.236
3	Technical consultant	10–14	30–39	18	0.164
4	Engineer	10–14	30–39	16	0.145
5	Technical consultant	≥20	≥50	26	0.236
Aggregate	-	-	-	110	1

**Table 14:** Prior and posterior probabilities and ROV values of the DBN model.

Node Symbol	State	Expert 1	Expert 2	Expert 3	Expert 4	Expert 5	FPS	Prior Probability (FPR)	Posterior Probability	RoV
D1-1	0	VL	VL	L	VL	VL	0.100454	1.67 × 10^−5^	7.47 × 10^−5^	3.480962
D1-2	0	L	ML	ML	ML	L	0.285764	7.54 × 10^−4^	3.37 × 10^−3^	3.476295
D2-1	0	L	L	ML	L	L	0.227114	3.46 × 10^−4^	-	-
D2-1	2	ML	L	L	L	L	0.231270	3.68 × 10^−4^	3.44 × 10^−1^	933.8836
D2-2	0	L	ML	L	ML	L	0.258650	5.39 × 10^−4^	-	-
D2-2	2	L	ML	ML	L	L	0.259672	5.46 × 10^−4^	5.11 × 10^−1^	934.0998
D2-3	0	VL	L	L	L	L	0.175351	1.40 × 10^−4^	1.31 × 10^−1^	934.3830
D3-1	0	ML	L	VL	L	L	0.209731	2.63 × 10^−4^	4.51 × 10^−4^	0.718002
D4-1	0	L	VL	ML	L	ML	0.234015	3.83 × 10^−4^	4.05 × 10^−4^	0.057215
D4-2	0	VL	L	L	VL	VL	0.127423	4.27 × 10^−5^	4.51 × 10^−5^	0.057216
D5-1	0	L	VL	L	VL	L	0.153317	8.57 × 10^−5^	8.57 × 10^−5^	0.000584
D6-1	0	VL	L	VL	VL	L	0.132234	4.92 × 10^−5^	4.93 × 10^−5^	0.000836
D6-2	0	VL	L	L	VL	VL	0.127423	4.27 × 10^−5^	4.27 × 10^−5^	0.000835
D6-3	0	L	VL	L	L	L	0.174322	1.37 × 10^−4^	1.37 × 10^−4^	0.000832
D7-1	0	M	L	MH	ML	L	0.369974	1.79 × 10^−3^	1.79 × 10^−3^	0.000017
D7-2	0	MH	ML	M	M	ML	0.465746	3.90 × 10^−3^	3.90 × 10^−3^	0.000005
D7-3	0	VL	L	VL	L	VL	0.126581	4.16 × 10^−5^	4.17 × 10^−5^	0.001692
D7-4	0	L	ML	VL	ML	L	0.236981	4.00 × 10^−4^	4.01 × 10^−4^	0.001697
D7-5	0	ML	L	ML	VL	L	0.237538	4.03 × 10^−4^	4.03 × 10^−4^	0.000001
D8-1	0	L	ML	L	L	L	0.232559	3.75 × 10^−4^	4.65× 10^−4^	0.239061
D8-2	0	L	ML	L	ML	L	0.258650	5.39 × 10^−4^	6.68 × 10^−4^	0.239336
D9-1	0	VL	L	L	VL	L	0.154362	8.78 × 10^−5^	8.92 × 10^−5^	0.015756

**Table 15:** Expert evaluation results and state transfer probabilities for node D1-1.

Time (t)	Time (t − 1)			
	State 0		State 1	
	Expert Evaluation	Probability	Expert Evaluation	Probability
0	-	0.999994	L, ML, ML, L, M	1.17 × 10^−3^
1	VL, VL, VL, VL, VL	5.66 × 10^−6^	-	0.998833

**Table 16:** Expert evaluation results and conditional probabilities for node D1.

D1-1	D1-2	D1			
		Expert Evaluation	State 0	Expert Evaluation	State 1
0	0	-	0.999994	VL, VL, VL, VL, VL	5.66 × 10^−6^
	1	-	0.999994	VL, VL, VL, VL, VL	5.66 × 10^−6^
1	0	-	0.998993	ML, L, M, ML, L	1.01 × 10^−3^
	1	VL, VL, VL, VL, VL	5.66 × 10^−6^	-	0.999994

## Data Availability

Data available on request from the authors.
